# Multi-level analysis reveals the association between diabetes, body mass index, and HbA1c in an Iraqi population

**DOI:** 10.1038/s41598-022-25813-y

**Published:** 2022-12-07

**Authors:** Rasoul Kowsar, Alireza Mansouri

**Affiliations:** 1 Department of Animal Sciences, College of Agriculture, Isfahan University of Technology, Isfahan, 84156-83111 Iran; 2 Global Agromedicine Research Center (GAMRC), Obihiro University of Agriculture and Veterinary Medicine, Obihiro, Hokkaido Japan

**Keywords:** Metabolic disorders, Applied mathematics, Scientific data

## Abstract

Type 2 diabetes (T2D) known as a complex metabolic disorder may cause health problems and changes in blood biochemical markers. A growing number of studies have looked into several biomarkers and their connections with T2D risk. However, few have explored the interconnection of these biomarkers, as well as the prospective alterations in the diabetes biomarker correlation network. We conducted a secondary analysis in order to introduce a multi-level approach to establish a relationship between diabetes, pre-diabetes, blood biochemical markers, age, and body mass index (BMI). The dataset was obtained from the Mendeley Data (available at https://data.mendeley.com/datasets/wj9rwkp9c2/1. In this study, three groups were established: non-diabetic (n = 103), pre-diabetic (n = 53), and diabetic (n = 844). According to the Heatmap analysis, non-diabetic and pre-diabetic individuals had the lowest BMI, age, and HbA1c. Diabetes and pre-diabetes were correlated with BMI (r = 0.58 and − 0.27, respectively), age (r = 0.47 and − 0.28, respectively), and HbA1c (r = 0.55 and − 0.21, respectively) using Pearson analysis. Using multivariate analysis, we found that diabetes, BMI, age, HbA1c, cholesterol, triglyceride, LDL, VLDL, and HDL were all associated. Network analysis revealed a connection between BMI and diabetes at the highest cut-off point. Moreover, receiver operating characteristic (ROC) analysis validated the network findings, revealing that BMI (area under the ROC curve, AUC = 0.95), HbA1c (AUC = 0.94), and age (AUC = 0.84) were the best predictors of diabetes. In conclusion, our multi-step study revealed that identifying significant T2D predictors, such as BMI and HbA1c, required a series of mathematical analyses.

## Introduction

The number of people living with type 2 diabetes mellitus (T2D) has grown to 350 million worldwide^1^, and it is expected that this metabolic disease will grow to 592 million (1 in 10 adults) by 2035^2^. The number of adults with diabetes is expected to rise by 20% in developed countries and 70% in developing countries over the next 20 years^3^. Mainly, T2D is distinguished by hyperglycemia, or a persistently elevated glucose level in the blood^4^. The most serious complication in T2D is the severe increase in blood glucose levels, known as "Diabetic Hyperglycemia", which is usually caused by insulin hormone production and secretion deficiency, insulin function failure, or a combination of these two pathological disorders^5^. This syndrome is also characterized by a decrease in antioxidants, an abnormal metabolic pattern of lipids^6,7^, carbohydrates^8,9^, proteins^10^ and electrolytes^11^, as well as a change in the hepatic enzymes^12^.

Type 2 diabetes is a complex metabolic disease that can lead to health problems in the body^11^. A plethora of evidence illustrated the impact of hyperglycemia on various body tissues as a result of protein glycation^12^. The steady elevation of glycated hemoglobin (HbA1c) due to diabetes-related hyperglycemia, for example, is attributed to structural and functional modifications in the hemoglobin molecule^12^. The impact of hyperglycemia on blood indices does not exhibit any pathological symptoms, but it may be the cause of various adverse findings and chronic problems in diabetic patients^12^. Increased triglycerides (TGs), decreased high-density lipoproteins (HDL)^4^, increased very-low density lipoprotein (VLDL), and low-density lipoproteins (LDL) are some of the lipoprotein anomalies associated with T2D^6,13^. Insulin resistance is often associated with certain lipoprotein anomalies^6^. Insulin resistance can also be caused by genetics^14^, obesity, abdominal obesity^15^, physical inactivity^16^, and age^17^. Furthermore, increased TGs, low HDL, and hyperuricemia are important metabolic risk factors in people with insulin resistance^18^. Insulin resistance enhances the likelihood of having decreased glucose tolerance and T2D^18^.

Obviously, a long family history of T2D, age, obesity, and physical inactivity distinguish those at greatest risk^18^. The risk of diabetes has been shown to rise in lockstep with increasing baseline BMI levels, with each kg/m^2^ increase in BMI resulting in a 23% increase in T2D risk^19^. There is a linear link between BMI and the prevalence of T2D in all age groups, with a larger relationship in younger persons^19^.

We are typically concerned about what factors are connected with various outcomes or the strength of the relationship between a variable and an outcome, as well as between variables. There is definitely collinearity and complex interactions between biological indices that cannot be identified by basic mathematical analysis^20^. Typically, regression analysis is used to investigate such correlations. The downside of this strategy is that it makes certain assumptions, such as the outcomes and variables being completely independent of one another^21^. As a result, statistical significance is overstated^21^. Multilevel analysis, as one analytical method, may assist in resolving this difficulty by permitting the simultaneous assessment of group-level and individual-level aspects^22^. Multilevel analysis allows for the testing of more relevant hypotheses, particularly those pertaining to variance in outcomes or variable interactions^21^. For example, the principal component analysis (PCA) may be used to assess complex interactions, as well as the degree and direction of associations between variables and outcomes^23–25^. The ANOVA tests, in fact, are incapable of dealing with complicated treatment structures and multidimensional data, such as omics data^26^. Furthermore, network analysis can be used to determine central nodes that are linked to a certain outcome. For example, using network analysis, Santiago et al.^27^ showed that an inflammatory-related gene, Forkhead box O3 (FOXO3, as a major transcriptional regulator), is up-regulated in the blood of children with T2D and mild cognitive impairment. Using a correlation network, Huang et al.^28^ demonstrated the importance of the leptin system in the development of diabetes. Liu et al.^29^ stated that network-based analysis allows us to have a better understanding of the pathophysiology of T2D patients. They also emphasized the need of using a system biology approach to investigate complex disorders^29^. As a result, network analysis and PCA may offer a deeper understanding of the relationships between variables (such as blood indicators) and outcomes (i.e., diabetes).

The receiver operating characteristic (ROC) curve analysis was also used in this study. This method is extensively used in biomedical studies to measure how well medical diagnostic tests (or systems) can distinguish between two patient/health conditions^30^. In this approach, patient/health statuses are usually referred to as "diseased" and "non-diseased" based on test findings, and the optimum cut-off value with the highest diagnostic performance is established^30^. For example, the glutamine/glutamic acid ratio has been shown to be the best biomarker (the area under the ROC curve, AUC, 0.74) for predicting diabetic retinopathy in T2D patients^31^.

Due to the limited amount of evidence, we carried out a secondary multilevel analysis in this study to uncover potential relationship between some blood biochemical indices, age, and BMI in diabetic and pre-diabetic persons, as well as the best predictor of diabetes diagnosis.

## Methods

### Data collection

The dataset was obtained from the Mendeley Data (available at https://data.mendeley.com/datasets/wj9rwkp9c2/1)^32^. The data were gathered from the Iraqi population, as they were retrieved from the laboratory of Medical City Hospital and the Specialized Center for Endocrinology and Diabetes-Al-Kindy Teaching Hospital. Data was collected from patients' files and entered into the database to create the diabetes dataset. The protocol and methods were performed in accordance with the guidelines and regulations approved by the Committee on the ethics of the medical experiments of the Specialized Center for Endocrinology and Diabetes-Al-Kindy Teaching Hospital. The dataset included fasting blood glucose, age, gender, creatinine (Cr), body mass index (BMI), blood urea nitrogen (BUN), fasting lipid profile (including total, LDL, VLDL, TG, HDL and cholesterol), and HbA1c. The patient's diabetes disease classes were non-diabetes, pre-diabetes, and diabetes. At each visit, fasting venous blood samples were taken after at least a 10-h fast. Diabetes, pre-diabetes, and non-diabetes were identified as fasting plasma glucose levels of > 7.0 mmol/L, 5.5–7.0 mmol/L, and 5.5 mmol/L, respectively.

Serum TG, total cholesterol, LDL and HDL were measured on the AU 5800 autoanalyzer (Beckman, USA). On an autoanalyzer (Beckman 5800, USA), plasma glucose levels were analyzed using the glucose oxidase method. Body weight was assessed to the nearest 0.1 kg when wearing loose clothes with no shoes. The height was calculated to the nearest 0.1 cm. Body mass index was calculated by dividing one's weight in kilograms by one's height in meters squared.

### Data analysis

A total of 1000 patients were divided into three categories based on their diabetes risk: (1) diabetic (n = 843), (2) pre-diabetic (n = 53), and (3) non-diabetic or normal (n = 103) classes. Using the Anderson–Darling test, data was found to be normally distributed. The Pearson correlation (Bonferroni correction) was used in the bivariate study to determine the relationship between blood biochemical parameters, age, BMI, and diabetes incidence. To evaluate the validity of the relationships in the correlation matrix, the Bonferroni correction was used^33^.

Principal component analysis was used to assess the complicated relationship or multi-collinearity between blood biochemical parameters, age, BMI and the diabetes incidence. This strategy can be used to reduce the number of variables while retaining the majority of the essential information^34^. In this study, multiple data dimensions were reduced to two dimensions and the biplot was generated using PAST software. Correlations in the PCA biplot are expressed as directional vectors and determined by the angle between vectors^35^. Vectors with < 45° angles had a positive correlation, vectors with perpendicular angles (approaching 90°) had no relationship, and vectors pointing in opposite directions (approaching 180°) indicated a negative association.

The Heatmapper (http://www1.heatmapper.ca/expression/) was used to produce patterns of blood biochemical parameters, age, and BMI in relation to diabetes incidence^33^. The heatmap was created using the Pearson distance measure and complete linkage as the clustering method. Because the data were normally distributed, the Pearson distance metric was applied. In the network analysis, the Pearson similarity index was used in conjunction with the Fruchterman–Reingold algorithm as a force-directed layout algorithm^36^. This algorithm generates a network based on the frequency with which nodes are connected. Pearson correlation, PCA, and network visualization investigations were carried out using the PAST tools (available at: http://folk.uio.no/ohammer/past).

The ROC curve analysis was conducted on the basis of network outputs that defined a correlation between blood biochemical parameters, age, and BMI, and diabetes incidence using the easyROC web-tool (available at: http://www.biosoft.hacettepe.edu.tr/easyROC/)^37^. This was accomplished to determine the predictive strength and cut-off point of network-detected parameters for detecting diabetes occurrence using the AUC analysis. The optimal cut-off was determined by maximizing the Youden index^37^.

## Results

### Data characteristics

Table 1 shows the patient’s features. The non-diabetic group was 25–77 years old (44.2 ± 9.4, mean ± SD), the pre-diabetic group was 30–55 years old (43.3 ± 7.8), and the diabetic group was 20–79 years old (55.3 ± 7.5). In the non-diabetic, pre-diabetic, and diabetic classes, the percentage of males was 37.9, 67.9, and 58.1%, respectively. The mean (± SD) of BMI was 22.4 ± 1.4, 23.9 ± 2.7, and 30.8 ± 4.3 kg/m^2^ for non-diabetic, pre-diabetic, and diabetic classes, respectively. Bays et al.^38^ found that more than 75% of diabetic persons had a BMI more than 25 kg/m^2^.

**Table 1:** Data and patients characteristics.

Items	All patients, n = 1000	Non-diabetes, n = 103	Pre-diabetes, n = 53	Diabetes, n = 843
Age, years (mean ± SD)	53.5 ± 8.8	44.2 ± 9.4	43.3 ± 7.8	55.3 ± 7.5
Male, n/total n	565/1000 (56.5%)	39/103 (37.9%)	36/53 (67.9%)	490/843 (58.1%)
BMI, kg/m^2^	29.6 ± 5.0	22.4 ± 1.4	23.9 ± 2.7	30.8 ± 4.3
HbA1c, %	8.28 ± 2.53	4.56 ± 0.92	6.00 ± 0.19	8.88 ± 2.26
Chol, mmol/L	4.86 ± 1.30	4.27 ± 1.28	4.58 ± 1.04	4.95 ± 1.30
TG, mmol/L	2.35 ± 1.40	1.63 ± 1.03	2.13 ± 1.06	2.45 ± 1.43
HDL, mmol/L	1.20 ± 0.66	1.23 ± 0.51	1.13 ± 0.38	1.21 ± 0.69
LDL, mmol/L	2.61 ± 1.12	2.63 ± 0.98	2.49 ± 0.87	2.62 ± 1.14
VLDL, mmol/L	1.85 ± 3.66	0.94 ± 1.48	0.98 ± 0.50	2.02 ± 3.93
BUN, mmol/L	5.12 ± 2.94	4.68 ± 2.52	4.51 ± 2.02	5.22 ± 3.02
Cr, µmol/L	68.94 ± 60.0	62.80 ± 30.02	66.08 ± 41.57	69.87 ± 63.58

*n* number, *Cr* creatinine, *Chol* cholesterol, *TG* triglyceride, *BMI* body mass index.

**Figure 1:**
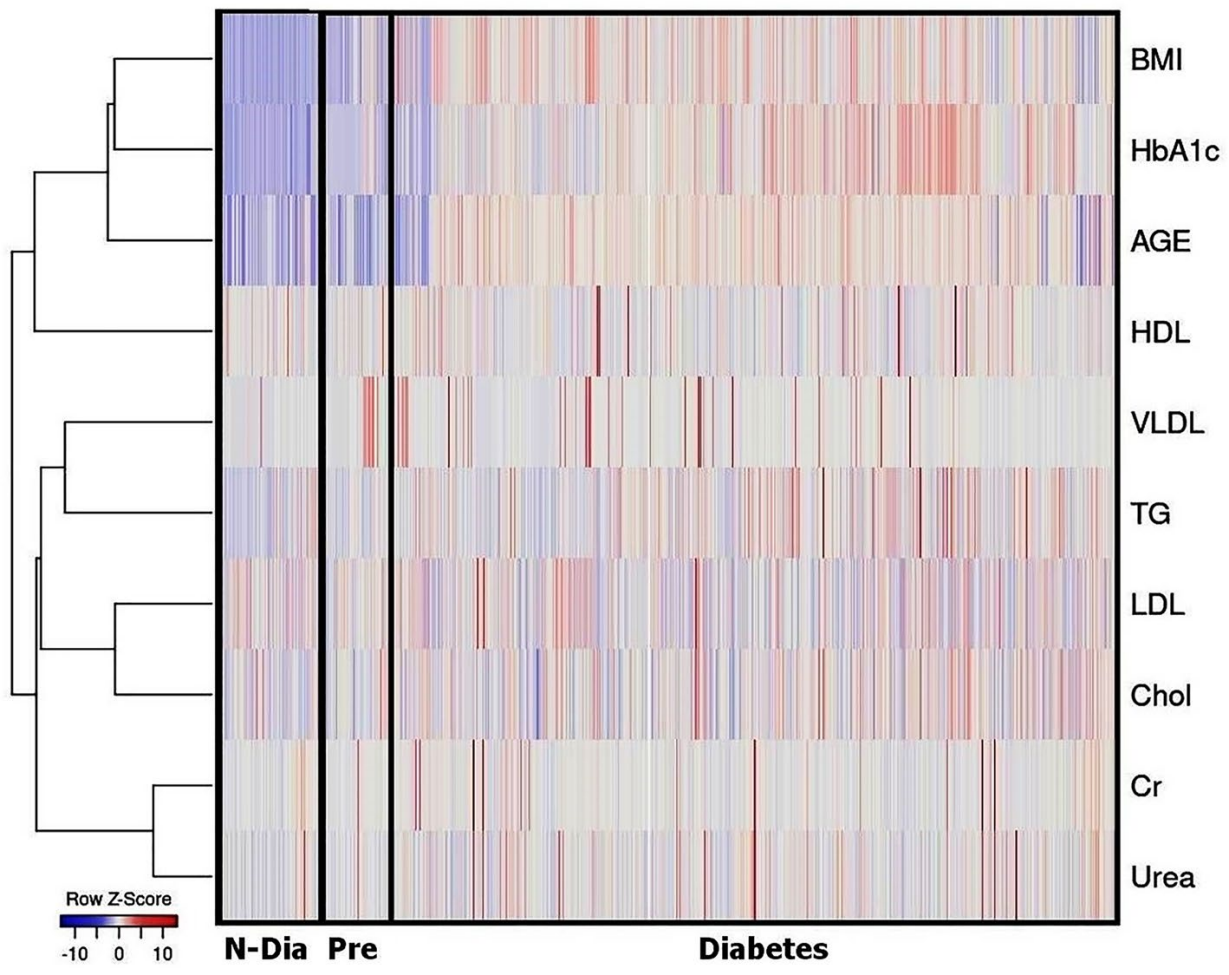
The heatmap of the blood biochemical markers, age, BMI across various groups. Heatmap was generated by “Heatmapper web tool”. The heatmap was created using the Pearson distance measure. Red represents higher values. Blue represents smaller values. The color intensity indicates the variation of the values in the color scale on the left side of the heatmap. *Dia* diabetes, *Pre* pre-diabetes, *N-Dia* non-diabetes, *Cr* creatinine, *Chol* cholesterol, *TG* triglyceride, *BMI* body mass index.

Figure 1 shows the pattern of data distribution of treatment groups. The HbA1c, cholesterol, TG, VLDL, BUN, and Cr levels were greater in the diabetic group compared to the pre-diabetic and non-diabetic groups. The pre-diabetic group had higher levels of HbA1c, cholesterol, TG, and Cr than the non-diabetic group (Table 1, Fig. 1). Other studies found that patients with T2D had higher levels of serum cholesterol, LDL, TG, creatinine, and BUN (above 25 mg/dL), as well as lower levels of HDL^39–42^.

### The association of blood biochemical markers, age, and BMI with diabetes

**Figure 2:**
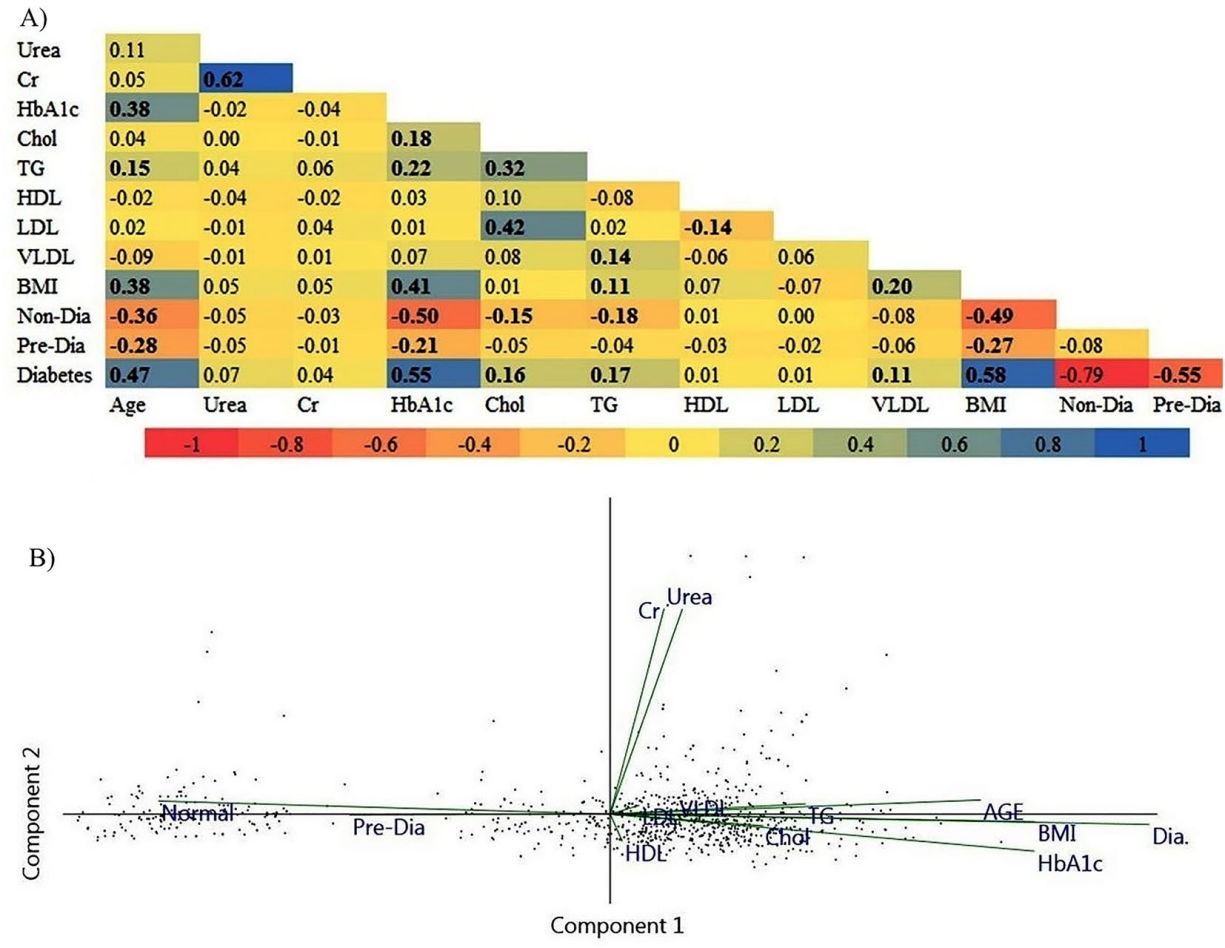
(**A**) Pairwise-Pearson correlation between blood biochemical markers, BMI, age and diabetes incidence. The color intensity is proportional to the correlation coefficients. The scale bar is the color range for different R values. Correlations that are significant at P < 0.05 are shown in the bold profiles. (**B**) Biplot of PCA derived from the blood biochemical markers, age, BMI, and diabetes incidence. Vectors with close angles (< 45°) indicate a strong correlation, vectors that are perpendicular indicate no correlation, and vectors in opposite directions (approaching 180°) indicate a negative correlation. *Dia* diabetes, *Pre-Dia* pre-diabetes, *Normal* non-diabetes, *Cr* creatinine, *Chol* cholesterol, *TG* triglyceride, *BMI* body mass index.

Diabetes was positively associated with BMI (r = 0.58), HbA1c (r = 0.55), and age (r = 0.47), according to the Pearson correlation (Fig. 2A). BMI was negatively associated with both pre-diabetes (r = − 0.27) and non-diabetes (r = − 0.49) groups. The pre-diabetes (r = − 0.21) and non-diabetes (r = − 0.50) groups showed a negative correlation with HbA1c. The Pearson correlation study revealed a relationship between HbA1c and age (r = 0.38) or BMI (r = 0.41, Fig. 2A).

The Pearson correlation analysis revealed a link between HbA1c and either cholesterol (r = 0.18) or TG (r = 0.22, Fig. 2A). According to the Ghari Arab et al. study^43^, there was a substantial link between HbA1c level and serum cholesterol, TG, LDL, and FBS; hence, a high level of HbA1C was related to dyslipidemia. Another study revealed no correlation between HbA1c and age, BMI, cholesterol, LDL, or HDL values^44^.

The PCA study revealed that there was multi-collinearity between HbA1c, cholesterol, TG, VLDL, LDL, BMI, age, and diabetes (Fig. 2B). According to the PCA study, the first four principal axis factors accounted for a reasonable amount of overall variance (58.2%). Oujidi et al.^45^ found a significant connection between age and HbA1c in T2D patients using a PCA approach.

**Figure 3:**
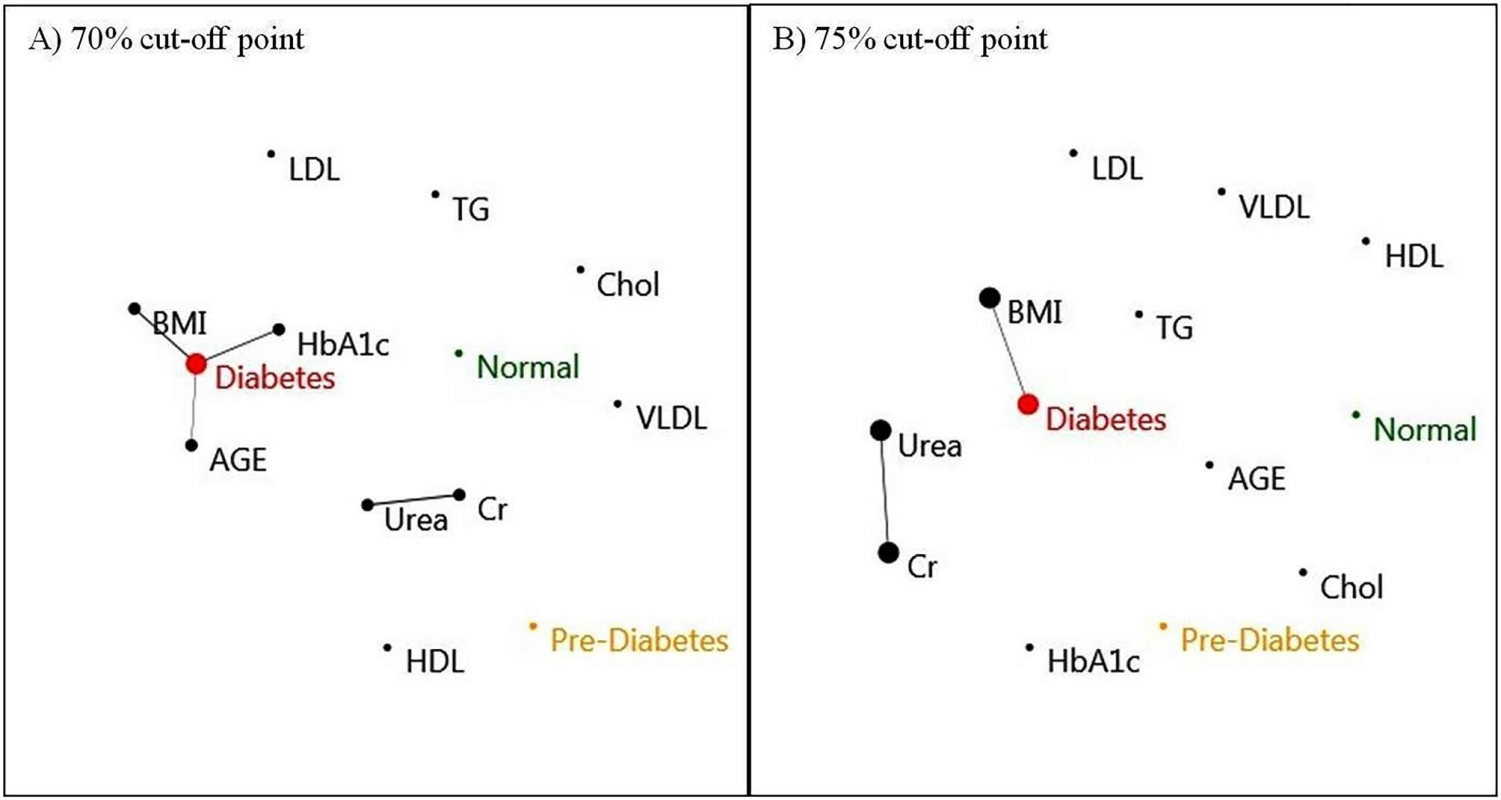
Correlation-based network analysis of blood biochemical markers, age, BMI, and diabetes. All parameters are defined by circles within the network. Network analysis and visualization was carried out using PAST and Fruchterman–Reingold algorithm as a force-directed layout algorithm. The Pearson correlation levels of (**A**) 70%, (**B**) 75% were selected to determine the connections between edges and nodes in terms of incident diabetes. The 70% and 75% cut-off points were the maximum level at which biochemical markers and all parameters demonstrated no relation with diabetes, respectively. Nodes represent parameters related to incident diabetes. Edges indicate the interactions between all factors. The node and edge sizes are proportional to the clustering and correlation coefficients, respectively. Small nodes and thin edges refer to small values. *Normal* non-diabetes, *Cr* creatinine, *Chol* cholesterol, *TG* triglyceride, *BMI* body mass index.

More specifically, network analysis showed that diabetes was associated with BMI, HbA1c, and age at the 70% cut-off point (this cut-off point was the maximum threshold that after that the biochemical markers had no association with diabetes, Fig. 3A). Network analysis revealed that, at the maximum cut-off point (75%), where there was no correlation between all parameters thereafter, diabetes incidence was only associated with BMI (Fig. 3B). Using network analysis, Huang et al.^28^ identified a link (positive correlation) between BMI and HbA1c.

### ROC analysis to identify best indicators of diabetes incidence

**Figure 4:**
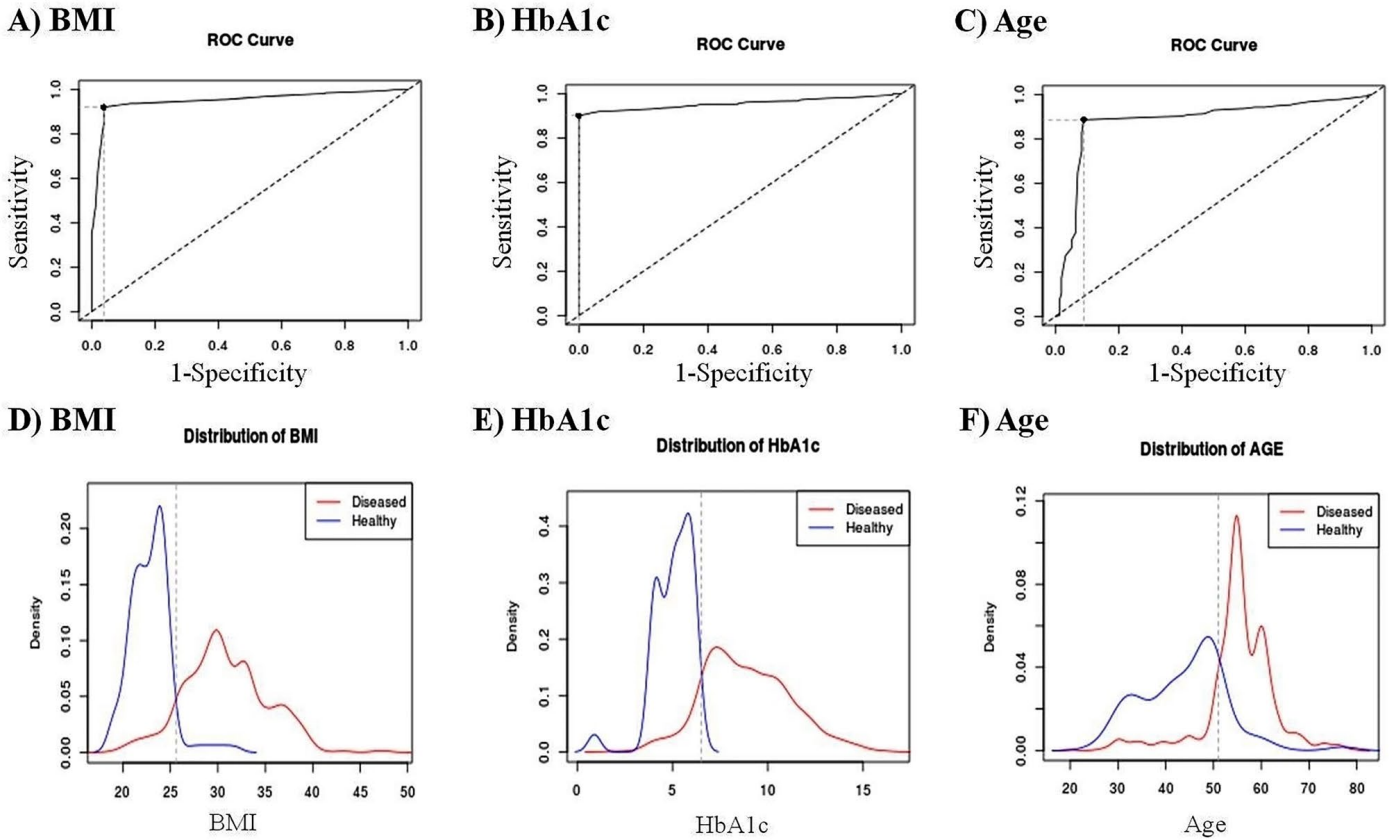
(**A**–**C**) The ROC curve of BMI, HbA1c, and age in predicting diabetes incidence. (**D**–**F**). The area under the ROC curve (AUC) is used to determine the best cut-off values for detecting incident diabetes.

Tables 2 and 3 demonstrate the AUC analysis for blood biochemical markers, BMI, and age in identifying diabetes patients. As seen in Table 3 and Fig. 4, the ROC analysis revealed that BMI (AUC: 0.95, p = 0.000), HbA1c (AUC: 0.94, p = 0.000), and age (AUC: 0.84, p = 0.000) had a strong predictive capacity to detect diabetic patients. According to the AUC analysis, BMI > 25.6 kg/m^2^, HbA1c > 6.5%, and age > 51 years were the cut-off values for identifying diabetes. Similarly, Tankova et al.^46^ demonstrated superior performance of HbA1c for detecting diabetes (AUC: 0.95) and pre-diabetes (AUC: 0.73) using ROC analysis.

**Table 2:** ROC analysis to describe the predictive power of the blood biochemical markers, age, and BMI to determine diabetes incidence.

Marker	AUC	SE	Lower limit	Upper limit	z	p value
BMI	0.95	0.01	0.93	0.97	45.70	0.00
HbA1c	0.94	0.01	0.91	0.96	36.66	0.00
Age	0.84	0.02	0.81	0.87	21.61	0.00
TG	0.64	0.03	0.59	0.69	5.58	0.01
Chol	0.63	0.02	0.58	0.67	5.27	0.01
VLDL	0.60	0.03	0.54	0.66	3.37	0.04
Urea	0.56	0.03	0.51	0.61	2.34	0.18
Cr	0.53	0.03	0.48	0.59	1.14	0.25
LDL	0.51	0.03	0.46	0.56	0.36	0.72
HDL	0.51	0.03	0.45	0.56	0.24	0.81

*Cr* creatinine, *Chol* cholesterol, *TG* triglyceride, *BMI* body mass index.

**Table 3:** ROC analysis to estimate the optimal cut-off point for the blood biochemical markers, age, and BMI to determine diabetes incidence.

Items	AUC	*p* value	Optimal cut off point	Sensitivity	Specificity	Positive predictive value	Negative predictive value	Positive likelihood ratio (LR)	Negative likelihood ratio (LR)	AUC rank
BMI, kg/m^2^	0.95	0.000	25.6	92.1	96.2	99.2	69.1	23.94	0.08	1
HbA1c, %	0.94	0.000	6.50	90.2	100	100	65.3	Infinity	0.09	2
Age, years	0.84	0.000	51.0	88.6	91.0	98.2	59.7	9.87	0.12	3

The analysis was carried out by determining the predictive power and cut-off point of the objects with the highest AUC (i.e., ≥ 80) for the detection of diabetes incidence. The optimal cut-off was calculated by maximizing the Youden index. *BMI* body mass index.

## Discussion

This multi-step study revealed that BMI, HbA1c, and age were all associated with diabetes and had the highest predictive potential. Surprisingly, none of them was associated with pre-diabetes. Furthermore, utilizing this multi-step approach, it was revealed that BMI was the most associated factor of diabetes, as demonstrated by network and ROC analysis. The data showed that identifying the most interconnected parameter, particularly in clinical examinations, required a multi-step strategy.

The presented approach, which included bivariate Pearson analysis, network analysis, and ROC analysis, revealed that BMI was the best predictor of diabetes. The prevalence of T2D has risen exponentially in recent decades as a result of lifestyle changes^19^. Obesity almost doubled worldwide between 1980 and 2008^47^. In 2008, there were over 200 million obese men and over 300 million obese women worldwide, accounting for 11% of adults^47^. Also, BMI has been linked to an increase in metabolic disorders such as T2D^19^. A meta-analysis of data found a greater correlation between BMI and diabetes risk in people under the age of 60^48^. According to the Bays et al. study^38^, T2D prevalence increased with increasing BMI. They found^38^ that more than 75% of T2D patients had a BMI greater than 25 kg/m^2^. In our research, the AUC analysis revealed that the cut-off point for diabetes occurrence was 25.6 kg/m^2^, which was consistent with the findings of Bays et al. study^38^. The current data indicated that BMI was closely correlated with diabetes and should be kept under surveillance.

The multiple factor analysis, PCA, revealed a complex collinearity between diabetes and factors used in this research. Data revealed a link between LDL, cholesterol, TG, VLDL, BMI, age, HbA1c, and diabetes. This suggested that, in addition to the best predictor uncovered by the current method, other lipid-related elements (such as LDL, cholesterol, TG, and VLDL) should be considered. Klisic et al.^49^ indicated that an undesirable lipid profile predicted HbA1c level in T2D patients. They advocated early recognition of dyslipidemia (an excess of lipids in the blood, such as TG, cholesterol, and fat phospholipids), as well as regulating and maintaining optimal lipid balance, as a precautionary tool for diabetes diagnosis^49^. Furthermore, Bays et al.^38^ reported that higher BMI was associated with a rise in the incidence of diabetes and dyslipidemia. Adipocyte hypertrophy, visceral adipose tissue formation, and lack of physical activity in genetically and environmentally vulnerable patients have been linked to metabolic disorders such as T2D and dyslipidemia^38^. Insulin resistance is believed to be the underlying cause of dyslipidemia in T2D patients^44,50^. Increased TG levels in T2D patients are caused by insufficient insulin production or impaired insulin action^44,50^. As a result of increasing substrate levels for TG synthesis, decreased insulin sensitivity causes increased hepatic VLDL production as well as late clearance of TG-rich lipoproteins^44,50^. Our findings revealed that an unfavorable lipid profile was also linked to diabetes, BMI, and HbA1c.

The current findings showed that HbA1c had a high potential to diagnose diabetes (AUC: 0.95). HbA1c has long been recognized as a glycemic control marker^49^. HbA1c appears to be a helpful, practical, and reliable method for identifying people with pre-diabetes and diabetes, according to the Tankova et al. research^46^. As a result, HbA1c monitoring should be included in the establishment of diagnostic techniques. It has been shown that each 1% reduction in HbA1c reduced the risk of diabetes-related complications by 37% and the risk of diabetes-related mortality by 21%^2^.We found that 6.5% HbA1c was the cut-off point for diabetes diagnosis, which was consistent with the WHO recommendation of 6.5% HbA1c as the cut-off point for diagnosing diabetes^51,52^. Because HbA1c represents mean plasma glucose over the previous 8–12 weeks, it may be used as both a diagnostic and a screening test for diabetes^53^. Furthermore, the Pearson correlation and PCA analysis revealed a relationship between HbA1c and BMI. It has been demonstrated that BMI is associated with poor glycemic control, as evidenced by a high HbA1c level^47,49^. Babikr et al.^54^ reported a positive relationship between BMI and HbA1c, which is similar to our findings. As a result, BMI and HbA1c should be closely monitored because they can serve as a powerful predictor of T2D.

## Conclusions

In this study, we investigated the capacity of a multilevel analysis to assess complicated interactions, as well as the magnitude and direction of correlations between variables, such as blood indices, age, BMI, and the outcome (i.e., diabetes). Such connections are typically investigated using regression analysis or ANOVA tests, which assume that the outcomes/variables are independent of one another, resulting in an overstatement of statistical significance^21^. It should be noted that multilevel analysis is particularly flexible in managing missing data and decreasing data dimension to reduce data features into fewer components to assist in the visualization of patterns^25,55^. For example, using network analysis, we found BMI as the central node associated with diabetes as a specific outcome. Kebede^56^ found, using a multilevel analysis, that among the parameters evaluated, the hemoglobin level and weight of patients were linked with the CD4 count of patients with diabetes. We previously found that the proportion of blastocyst formation in bovines was connected with various blood indexes or follicular fluid CRP and bilirubin employing a multilevel analysis^37,57^. Our findings using bivariate analysis indicated that age, BMI, and HbA1c had the highest connection with diabetes in the current research. Following that, PCA analysis indicated that additional parameters, such as an unfavorable lipid profile, were also linked to diabetes and higher HbA1c levels. Finally, network and ROC analysis revealed that, of all studied parameters, BMI had the best predictive power for diabetes diagnosis.

Therefore, it is necessary to conduct clinical studies using as many parameters as possible to identify the most associated patient features with diabetes. The findings also suggested that when investigating the relationship between variables, a multi-step strategy be employed to determine the indices that are the most interrelated.

## Data Availability

Statistical summaries of data generated and analyzed for the present article are included in the published article. Further details are available from the corresponding author on reasonable request.
